# Evaluating the quality of antihypertensive drugs in Lagos State, Nigeria

**DOI:** 10.1371/journal.pone.0211567

**Published:** 2019-02-13

**Authors:** Elizabeth Thithi Ndichu, Kelechi Ohiri, Oluwafemi Sekoni, Olasunmbo Makinde, Kevin Schulman

**Affiliations:** 1 Duke Global Health Institute, Durham, North Carolina, United States of America; 2 Health Strategy and Delivery Foundation, Lagos, Nigeria; 3 Clinical Excellence Research Center, Department of Medicine, Stanford University, Stanford, California, United States of America; Makerere University School of Public Health, UGANDA

## Abstract

**Background:**

As the burden of noncommunicable diseases grows, access to safe medical therapy is increasing in importance. The aim of this study was to develop a method for evaluating the quality of antihypertensive drugs and to examine whether this prevalence varies by socioeconomic variables.

**Methods:**

We conducted a cross-sectional survey of registered pharmacies in 6 local government areas (LGAs) in Lagos State, Nigeria. In each LGA, we sampled 17 pharmacies from a list of all registered pharmacies derived from the Pharmacists Council of Nigeria. We assessed drug quality based on (1) the level of active pharmaceutical ingredients (APIs), which identified falsely labeled drug samples; and (2) the amount of impurities, which revealed substandard drug samples in accordance with the international pharmacopoeia guidelines. Good-quality drugs met specifications for both API and impurity.

**Results:**

Of the 102 drug samples collected, 30 (29.3%) were falsely labeled, 76 (74.5%) were substandard,78 (76.5%) were of poor quality and 24 (23.5%) were of good quality.Among the falsely labeled drugs, 2 samples met standards set for purity while 28 did not. Among the 76 substandard drug samples, 28 were also falsely labeled. Of the falsely labeled drugs, 17 (56.7%) came from LGAs with low socioeconomic status, and 40 (52.6%) of the substandard drug samples came from LGAs with high socioeconomic status. Most of the good-quality drug samples, 14 (58.3%), were from LGAs with low socioeconomic status. Eighteen (60%) of the falsely labeled samples, 37 (48.7%) of the substandard samples, and 15 (62.5%) of the good-quality drug samples were from manufacturers based in Asia. The average price was 375.67 Nigerian naira (NGN) for falsely labeled drugs, 383.33 NGN for substandard drugs, and 375.67 NGN for good-quality drugs. The prevalence of falsely labeled and substandard drug samples did not differ by LGA-level socioeconomic status (*P* = .39) or region of manufacturer (*P* = .24); however, there was a trend for a difference by price (*P* = .06).

**Conclusion:**

The prevalence of falsely labeled and substandard drug samples was high in Lagos. Treatment of noncommunicable diseases in this setting will require efforts to monitor and assure drug quality.

## Introduction

Poor-quality drugs are a global crisis posing a threat to global health and especially the treatment of noncommunicable diseases. Although the deleterious consequences of poor-quality drugs have been described, efforts to control the illicit trade of these drugs are lagging.[1],[2] Addressing the issue of poor-quality medicines is complex and requires coordinated collaboration of industry and government across the supply chain.[1],[3]

A 1992 convention of the World Health Organization (WHO) led to a definition of counterfeit medicines: “A counterfeit medicine is one which is deliberately and fraudulently mislabeled with respect to identity and or source. Counterfeiting applies to both branded and generic products. Counterfeit products may include products with the correct ingredients or with the wrong ingredients, without active ingredients, with insufficient active ingredients or with fake packaging.”[4] In 2012, the WHO revised the definition of poor-quality medicines under the clause Substandard, Spurious, Falsely Labelled, Falsified and Counterfeit (SSFFC).[3],[5] Other organizations, such as the US Food and Drug Administration (FDA), have defined substandard medicines as those that are contaminated, products containing the wrong or no active ingredient, stolen or diverted products, and expired, adulterated, or unapproved products.[6] In May 2017, the WHO replaced the SSFFC term with “substandard and falsified medical products,” following a request during the Seventieth World Health Assembly.[7] In this paper, we use the term *falsely labeled* to refer to drugs that have higher or lower amounts of the API than what is labeled on the product; we use *substandard* to refer to drugs with high levels of impurities, *good-quality drugs* for those that meet expectations for API and purity, and poor-quality drugs as those that are substandard or falsely labeled.

Poor-quality drugs enter the supply chain through any segment: manufacturers, distributors, and retailers.[8],[9],[10],[11] Most evaluations have focused on the retail segment.[12] Collected products were from formal facilities, such as pharmacies, distribution centers, and wholesalers. Informal facilities, such as open-air markets, unregistered drugstores, and online resources, have also been sampled.[13]

We describe a drug sampling strategy in the independent retail pharmacy segment in Lagos State, Nigeria. We focused on assessing the quality of medications for a common noncommunicable disease, hypertension.

## Materials and methods

### Study areas

We conducted the study in Lagos, Nigeria, a state divided into 20 local government areas (LGAs), which vary in population and size. We selected six LGAs for the study based on socioeconomic status and population. Data from the 2006 national census, the most recent population count, was acquired from the National Population Council of Nigeria (**Table 1**).These data were considered in the preliminary stages of the study design but were not used to identify the final six LGAs as they contained out-of-date information. LGAs were also categorized based on urbanization, structural development, and transport conditions, which we used as markers of socioeconomic status. In addition, the identification of LGAs was based on a consensus of the principal investigator at the Duke Global Health Institute and the expert opinion of Health Strategy and Delivery Foundation staff members. [14]

**Table 1:** Population of Lagos State, Nigeria, by Local Government Area in 1996 and 2006.

1996		2006	
Local Government Area	Population	Local Government Area	Population
Agege	790,333	Agege	461,743
Alimosho	522,854	Ajeromi-Ifelodun	687,316
Badagry	144,722	Alimosho	1,391,571
Epe	123,119	Amuwo-Odofin	328,975
Eti-Osa	213,199	Apapa	222,986
Ibeju/Lekki	30,259	Badagry	237,731
Ikeja	246,791	Epe	181,734
Ikorodu	224,089	Eti-Osa	283,791
Lagos Island	201,424	Ibeju/Lekki	117,793
Lagos Mainland	341,649	Ifako-Ijaye	427,737
Mushin	654,988	Ikeja	317,614
Ojo	1,256,167	Ikorodu	527,917
Oshodi/Isolo	545,777	Kosofe	682,772
Shomulo	935,789	Lagos Island	212,700
Surulere	715,859	Lagos Mainland	326,700
		Mushin	631,857
		Ojo	609,173
		Oshodi-Isolo	629,061
		Shomolu	430,569
		Surulere	502,865
Total population	6,947,019		9,113,605

### Sampling strategy

In this cross sectional study, we sampled nifedipine, a calcium channel blocker with a preferred strength of 20 or 30 mg. Calcium channel blockers are the most commonly used antihypertensive drugs and their availability is widespread in Lagos State. [15],[16],[17],[18],[19]

Samples of nifedipine tablets were collected between May and July 2017 from registered pharmacies using a stratified sampling approach. First, we obtained a comprehensive list of registered pharmacies in Lagos State and their location from the Pharmacists Council of Nigeria.[20] These included both chain and independent pharmacies. Second, we identified the registered facilities on the busiest streets in each LGA. All state- and federal-level hospitals in the LGAs were automatically included. We excluded chain pharmacy stores since the products they sell might all be sourced from the same distributors (and thus we would be significantly oversampling this element of the supply chain with our retail purchase design).

We only collected samples of branded medicines. In the initial sampling process, we observed that two main nifedipine brands were dispensed in most pharmacy stores. In order to diversify the samples collected, if multiple branded medicines were available, the least popular brand was purchased. Drugs sampled were to be well-labeled in sealed blister packages indicating the brand name, expiration date, and manufacturer name and address.

The survey investigator involved in the sampling process was a local resident of Lagos State trained on the sampling criteria before the sampling began. The criteria involved presenting to the randomly selected pharmacy as a shopper by posing as customer without identifying himself as an investigator, presenting a prescription, and requesting to purchase branded nifedipine 20 or 30 mg. The surveyor was expected to purchase the least common nifedipine drug sample. Following full payment for the drugs, the surveyor requested a receipt indicating the cost of the drug and name of the facility.

Data for all collected samples included date of collection, buying price, pharmacy name and location, and origin of the drug. Data were documented in a study database. Following the sampling process, each sample was kept in its original package and was placed in a transparent resealable plastic bag. A unique code derived from the sampling date, LGA, cost, and numerical value on the sample record list was assigned to each sample. This code was printed on adhesive labels that were placed on the plastic bag. Samples were stored securely in temperatures below 25 degrees Celsius. Upon completion of the sampling process, the samples were divided into six packages based on the LGA of origin. The six packages were further secured using bubble wrap for protection from mechanical stress. The drugs were transported via courier service to an FDA-registered laboratory in North Carolina, USA.

### Drug quality measures

Nifedipine samples were assessed for quality at Campbell University College of Pharmacy and Health Sciences laboratory in North Carolina using reverse-phase high-performance liquid chromatography (HPLC) with mass spectrometry. Before the analysis, the samples were visually inspected and examined for abnormalities such as broken blister packaging and passed expiration dates.

We followed recommended international pharmacopeia guidelines to assess drug quality. The guideline recommends that amounts of API in each nifedipine tablet should be between 90% and 110% of the labeled dose and that impurities in a nifedipine sample should be less than 2% for nifedipine nitrophenylpyridine analog and less than 0.5% for nifedipine nitrosophenylpyridine analog.[6] The level of API was used to identify falsely labeled samples, and the amount of impurities was used to identify substandard samples. Good-quality drugs met specifications for both the API and impurities.

### Statistical analysis

We summarized categorical variables using frequencies and percentages and continuous variables using means (standard deviation) or medians (interquartile ranges). We compared LGA-level socioeconomic characteristics, drug prices, and country of origin across drug quality measures using chi-squared test and Kruskal-Wallis test for categorical variables and Mann-Whitney-U tests for continuous variables.[21]

Logistic regression models were used to examine associations between three key predictors (LGA-level socioeconomic status, price of the drug, and geographic location of the manufacturer) and API amount and impurity levels. All hypothesis tests were two-sided at the 5% α level. Stata version 14.2 (Stata Corp., College Station, Texas, USA) was used for the statistical analysis.

### Ethical approval

A request for exemption from Institutional Review Board review was sought before commencement of the study from Duke University School of Medicine and College of Medicine of the University of Lagos, Health Research Ethics Committee. Further permission was sought from the US Food and Drug Administration for authorisation of importation of drug samples for laboratory evaluation.

## Results

Seventeen pharmacies were sampled from each of the six LGAs (**Table 2**). A total of 102 samples of nifedipine were collected from public and privately owned pharmacies. Of these, 101 (99.0%) were from private pharmacies. All pharmacies were registered with the Pharmaceutical Council of Nigeria. Drug prescriptions were not required for privately owned facilities and were needed only when we sought a drug sample from a state or federal facility. A total of 14 branded drugs were collected from the six LGAs, with each sample packet ranging between 15 and 30 tablets. In most pharmacies, only two branded types were available. The dosage of branded nifedipine collected was 20 mg (n = 94; 92.16%) and 30 mg (n = 8; 7.8%). None were expired at the time of purchase, and all were in blister packages except for one sample that was packed in a transparent resealable plastic bag.

**Table 2:** Registered pharmacies in Lagos State, Nigeria, by Local Government area.

Local Government Area	Registered Pharmacies, No.	Sampled Pharmacies
Alimosho	123	17
Mushin	63	17
Eti-Osa	90	17
Ikeja	128	17
Ikorodu	88	17
Ibeju Lekki	35	17

### Compliance with specifications

Based on inspection, all samples passed the pre-laboratory visual screening tests and were evaluated through the HPLC test. The drug samples came in blister packages except for one sample whose pills were in a plastic bag.Thirty samples (29.4%) were falsely labeled such that the label amount fell below the FDA and USP 90% lower limit (**Table 3**). One sample had label amounts exceeding the expected 110% upper limit: it contained 27 mg of nifedipine API, despite the 20 mg label.

**Table 3:** Percentage of Falsely labeled Drugs in Lagos State, Nigeria, by Local Government area and socioeconomic status.

	Local Government Area	Quality Categories, No. (%)	
High socioeconomic status		Falsely Labeled (API < 90% or > 110%)	Correctly Labeled (API > 90 to < 110%)
	Eti-Osa	1 (5.9)	16 (94.1)
	Ibeju-Lekki	1 (5.9)	16 (94.1)
	Ikeja	11 (64.7)	6 (35.3)
Low socioeconomic status	Alimosho	7 (41.2)	10 (58.8)
	Ikorodu	8 (47.1)	9 (52.9)
	Mushin	2 (11.8)	15 (88.2)
	Total	30 (29.4)	72 (70.6)

Abbreviation: API, active pharmaceutical ingredient.

Nifedipine nitrophenylpyridine analog constituted the only impurity in the samples. It was found in amounts exceeding the 2.0% specification in 76 (74.5%) samples (**Table 4**). Of the 102 samples, 78 (76.5%) were poor quality drugs and 24 (23.5%) met both label amount and purity standards and were considered high-quality.

**Table 4:** Percentage of substandard drugs in Lagos State, Nigeria, by Local Government area and socioeconomic status.

Local Government Area	Quality Categories, No. (%)	
	Substandard (< 98%)	Meets Purity Standards (> 98%)
High socioeconomic status		
Eti-Osa	7 (41.2)	10 (58.8)
Ibeju-Lekki	16 (94.1)	1 (5.9)
Ikeja	17 (100.0)	0
Hard to reach		
Alimosho	16 (94.1)	1 (5.9)
Ikorodu	14 (82.4)	3 (17.7)
Mushin	6 (35.3)	11 (64.7)
Total	76 (74.5)	26 (25.5)

### Local government areas

Samples were collected from six LGAs. Among the 30 falsely labeled drugs, 17 (56.7%) came from LGAs with low socioeconomic status (**Table 3**). Of the 76 samples with high levels of impurities, 40 (53%) were from LGAs with high socioeconomic status. Six samples from Ikeja, an LGA with high socioeconomic status, had impurities in quantities exceeding 10% (**Table 4**). The highest proportion of good-quality drugs, 58.3%, was from an LGA with low socioeconomic status. The remaining good-quality drugs (41.7%) were from LGAs with high socioeconomic status. The difference in the number of poor-quality drugs between high and low socioeconomic status regions was not statistically significant (*P* = .35).

### Countries of origin

The samples analyzed in this study came from six countries, according to their labels: India, Israel, Nigeria, Switzerland, Germany, and Slovenia. We grouped these countries into continents. Almost half of the samples were labeled as coming from Asia (n = 50 [49.0%]). Thirty-nine (38.3%) of the samples were manufactured in Africa and came from manufacturers based in Nigeria. Seven (6.9%) were from manufactures based in Europe.We could not establish the location of manufacturers of 6 (5.9%) of the samples.

Overall, of the 30 (29.4%) falsely labeled samples, 18 (60%) were from manufacturers in Asia, 7 (23.3%) from Africa, and 5 (16.7%) from Europe or unknown locations (**Table 5**).

**Table 5:** Number of poor-quality drugs in Lagos State, Nigeria, based on location of drug manufacturer and Local Government area.

Location of Manufacturer	Poor-Quality Drugs, No. (No. Falsely Labeled, No. Substandard)						
	Alimosho	Mushin	Eti-Osa	Ikeja	Ikorodu	Ibeju-Lekki	Total
Asia	10 (6,10)	2 (0, 2)	1 (1, 1)	8 (6, 8)	7 (4, 7)	9 (1, 9)	37 (18, 37)
Europe	2 (0,2)	1 (1, 0)	0 (0, 0)	1 (1, 1)	1 (1, 1)	0 (0, 0)	5 (3, 4)
Africa	4 (1,4)	3 (0, 3)	6 (0, 6)	7 (3, 7)	4 (3, 4)	7 (0, 7)	31 (7, 31)
Unknown	0 (0,0)	1 (1, 1)	0 (0, 0)	1 (1, 1)	2 (0, 2)	0 (0, 0)	4 (2, 4)

Among the 76 (74.5%) poor-quality samples, 37 (48.7%) were manufactured in Asia. The second most common origin of substandard samples was Africa with 31 (40.8%). Good-quality drugs comprised 24 (23.5%) of the samples collected; 15 (62.5%) were manufactured in Asia, 8 (33.3%) in Africa, and 4 (16.7%) in Europe or unknown locations.

### Drug prices

The mean price of the samples was 380.30 NGN (1 US dollar is equivalent to 368 NGN). Good-quality drugs had a mean price of 431.74 NGN [SD, 219.55], while poor quality drugs had a mean price of 365.12 NGN [SD, 278.05]); the difference was not statistically significant (*P* = .29). The highest mean price came from an LGA with low socioeconomic status, which also had the highest number of good-quality drugs. The second highest prices were from an LGA with high socioeconomic status where samples failed to meet quality standards. The two lowest prices were from areas characterized as being both low and high social economic status and both had poor-quality drug samples amounting to over 80% of their samples. Overall, mean price in LGAs with high socioeconomic status was 382.80 NGN; [SD, 233.36] while in LGA’s with low socioeconomic status it was 377.84 NGN [SD, 297.51]); the difference was not statistically significant (*P* = .92).

### Predictors of drug quality

Only 24 (23.6%) samples met specifications for label amount and purity and were considered good-quality samples. Forty-eight (47.06%) samples met specifications for API but not purity, whereas 2 (2.0%) met purity standards but failed to satisfy the API standards. The samples that met the label amount standards and those that met the purity standards were not significantly different (*P* < .36) among high and low socioeconomic status LGAs. Samples meeting the label API standards did not differ by socioeconomic status (*P* = .35), price (*P* = .43), or region of manufacturer (*P* = .89). Similarly, label API and purity standards of the samples did not differ by LGA socioeconomic status (*P* = .39) and region of manufacturer (*P* = .24), but there was a trend for a difference by price of the drug samples (*P* = .06).

Compared to LGAs with high socioeconomic status, those with low socioeconomic status were 8% less likely to have poor-quality drugs: β –0.08 (95% CI, –0.24 to 0.09). Similarly, every unit increase in the price of drugs was associated with 5% lower quality of the drug: β –0.05 (95% CI, –0.16 to 0.07).

Drug quality did not differ significantly by region from which the drugs were manufactured. Compared to drugs manufactured in Africa, the quality of drugs from Asia (β–0.34; 95% CI, –0.22 to 0.15), Europe (β–0.08; 95% CI, –0.43 to 0.27), and unknown locations (β –0.12; 95% CI, –0.50 to 0.25) were similar.

## Discussion

Our report represents the first study of antihypertensive drugs in Lagos State, Nigeria. Of the 102 samples collected, 24 (23.5%) met API and purity standards following biochemical analysis. Although 70.6% of the samples met the standards for label amounts, the levels of impurities in the samples contributed to the large number of poor-quality drugs. A higher proportion of drugs with high levels of impurities came from areas with high socioeconomic status. Contrary to our expectations, a higher number of drugs that met all label amount and purity standards and were of good quality came from areas of low socioeconomic status. Good-quality drugs had a higher mean price compared to low-quality drugs, though the relationship was not significant (*P* = .29).

Other studies have explored the prevalence of poor-quality drugs and have argued for implementation of better surveillance of the pharmaceutical supply chain. For example, in a study evaluating seven routinely used cardiac drugs in 10 sub-Saharan Africa countries, 50% of the drugs produced in Asia and sold in street markets were of poor quality.[17] Our method is ideal for performing rapid surveys of the supply chain, particularly in resource-constrained settings. Mystery shoppers can help eliminate bias in the evaluation process and increase the demand for good products among consumers and suppliers. In addition, we performed biochemical analysis through HPLC, the gold standard in quantifying drug compounds. [22] More recent research has identified many different assay approaches to more quickly and reliably determine drug quality in the field. For example, chemical analysis methods such as colorimetric methods and quicker testing methods such as near infrared spectrometry can be used across all levels of the supply chain.[22] Another example of a successful time-efficient and affordable testing method is the Global Pharma Health Fund Minilab, which is a basic thin-layer chromatographic test. Other technologies are designed to ensure the integrity of the supply chain including formulating medicines with biodegradable barcodes and QR(Quick Response) codes,which are machine readable printed patterns containing information on a product.[1],[22],[23],[24]

This study reveals the challenges of assuring drug quality at the retail level where drug quality is impossible to determine from visual inspection alone.[25] Ensuring the availability of high-quality drugs will require robust efforts across the entire supply chain: manufacturers, distributors and retailers.

One of our most important findings is that price was not related to quality. Our findings suggest it is possible for manufacturers to supply high-quality products to this market at current market prices. This suggests that efforts to expand the supply of high-quality drugs is a solvable problem. Addressing the prevalence of low-quality drugs can use strategies based on a public regulatory approach, a private market approach, or a combination of the two.

The government of Nigeria could consider ways to enhance the role of preexisting regulatory bodies such as the National Agency for Food and Drug Control (NAFDAC), a federal agency under the Federal Ministry of Health, in assuring drug quality. Cross-border and multisector partnerships are likely to be beneficial as most drugs are imported into the region from Asia. We did not sample drugs at the manufacturing plant, but we found that drug quality varied by the manufacturer reported on the drug package. We collected 14 different brands. Good- and poor-quality drugs came from all brands. Sampling techniques aimed at assuring the quality of drugs at the manufacturer level could address poor quality before dissemination into the supply chain. Mislabeling of drugs and high levels of impurities can be addressed by ensuring better manufacturing practices. Without sampling at the manufacturer level, it is impossible to determine whether poor-quality products were manufactured by illicit drug vendors who use counterfeit packaging or the right raw materials that do not adhere to good manufacturing practices.

At the distributor level, identification of poor-quality drugs is a daunting process for most regulatory agencies. Poor- and high-quality drug samples in our study had similar packaging, rendering unaided visual inspection insufficient in assessing quality. Moreover, our assessment revealed that poor quality-drugs came from all regions and manufacturers. To ensure good-quality drugs are supplied to retail pharmacies, quality inspections such as batch testing products will be needed to assure the integrity of the supply chain. Development of business frameworks and practices that motivate distributors to assure the quality of drugs could improve the quality of products moving through the supply chain. CFW franchise network in Kenya is an example of a business model placed to assure the distribution of good-quality products through the pharmaceutical supply chain.[26]

Retail pharmacies are the last point of interdiction of poor-quality drugs before they reach patients. Incentives to reward supply chain security could help to address the prevalence of poor-quality drugs. With the private sector comprising a majority of retailers, retail pharmacies should be offered incentives to improve drug quality by assuring higher margins for the trade of high-quality products. One possible approach to enhancing the market for high-quality drugs is the use of mystery shoppers in rapid market surveys. Not only is this approach cost-effective and readily available, publicizing the results of the surveys could result in public pressure for higher-quality products. Government and private sector collaboration could raise awareness about the presence of poor-quality drugs.

Most reports, ours included, are limited in assessing drug quality since they only provide a snapshot of the market at a point in time.[18] This study was conducted in Lagos State, assessed one type of medication, and sampled registered pharmacies. The findings are not generalizable to the rest of the country, other products, chain stores, or unregistered facilities.

The list of registered facilities in the LGAs sampled was not up-to-date and did not provide precise location details. It was not possible to create an exhaustive list of all pharmacies, a necessary condition for comprehensive retail product sampling. Also, we did not sample the significant number of informal (unregistered) pharmacies that we observed. Thus, our assessment of the prevalence of poor-quality products could be conservative if drug quality is lower at unregistered facilities.We sought to compare the quality of drugs between government and private facilities. However, government facilities in two of the LGAs did not carry nifedipine. Finally, we assessed the q uality of drugs at independent pharmacies. Future efforts should evaluate drug quality at chain pharmacies.

We were not certain that countries of origin indicated on samples were the true sources, especially for drugs that failed to meet API and purity standards. Yet, manufacturers from Asia were a risk factor for poor-quality drugs. This finding is consistent with reports from the WHO and the United Nations Office of Drugs and Crime, which have reported that Asia accounts for the largest share in the trade of poor-quality medicines.[27],[28]

Despite these challenges, our study had many notable strengths, including the largest sample of drugs sourced from registered drugs facilities in Lagos State, the use of mystery shoppers to eliminate bias in sample collection, and the testing of samples in a reference laboratory using HPLC. The study’s simple stratified sampling methodology can be used as a reference point for other similar research projects.

## Conclusions

In a study of the quality of medications for a noncommunicable disease from retail pharmacies in Lagos State, Nigeria, we found a high prevalence of falsely labeled and substandard samples. The study suggests an urgent need to monitor and assure drug quality for populations across the pharmaceutical supply chain.

## Supporting information

S1 Data Complete raw dataset of the drug samples collected in Lagos State, Nigeria. Includes the drug samples’ price,amount of active pharmaceutical ingredient, purity level and socioeconomic status of local government area where the drug was sourced.
(CSV)
